# Cerebral haemodynamic response to somatosensory stimulation in preterm lambs is enhanced following sildenafil and inhaled nitric oxide administration

**DOI:** 10.3389/fphys.2023.1101647

**Published:** 2023-01-25

**Authors:** Ishmael Miguel Inocencio, Navneet Kaur, Nhi T. Tran, Flora Y. Wong

**Affiliations:** ^1^ The Ritchie Centre, The Hudson Institute of Medical Research, Melbourne, VIC, Australia; ^2^ Department of Paediatrics, Monash University, Melbourne, VIC, Australia; ^3^ Monash Newborn, Monash Children’s Hospital, Melbourne, VIC, Australia

**Keywords:** sildenafil, inhaled nitric oxide (iNO), preterm brain, cerebral haemodynamic functional response, neurovascular coupling (NVC)

## Abstract

**Background:** Neurovascular coupling (NVC) leads to an increase in local cerebral blood flow and oxygenation in response to increased neural activity and metabolic demand. Impaired or immature NVC reported in the preterm brain, potentially reduces cerebral oxygenation following increased neural activity, predisposing to cerebral tissue hypoxia. Endogenous nitric oxide (NO) is a potent vasodilator and a major mediator of NVC and the cerebral haemodynamic response. NO modulators, such as inhaled nitric oxide (iNO) and sildenafil, induce vasodilation and are used clinically to treat pulmonary hypertension in preterm neonates. However, their impact on NVC in the preterm brain are unknown. We aimed to characterise the cerebral functional haemodynamic response in the preterm brain exposed to NO modulators. We hypothesized that iNO and sildenafil in clinical dosages would increase the baseline cerebral perfusion and the cerebral haemodynamic response to neural activation.

**Methods:** Preterm lambs (126–7 days’ gestation) were delivered and mechanically ventilated. The cerebral functional haemodynamic response was measured using near infrared spectroscopy as changes in cerebral oxy- and deoxyhaemoglobin (ΔoxyHb, ΔdeoxyHb), following left median nerve stimulations of 1.8, 4.8, and 7.8 s durations in control preterm lambs (*n* = 11), and following 4.8 and 7.8 s stimulations in preterm lambs receiving either sildenafil citrate (*n* = 6, 1.33 mcg/kg/hr) or iNO (*n* = 8, 20 ppm).

**Results:** Following 1.8, 4.8, and 7.8 s stimulations, ∆oxyHb in the contralateral cortex increased (positive functional response) in 7/11 (64%), 7/11 (64%), and 4/11 (36%) control lambs respectively (*p* < 0.05). Remaining lambs showed decreased ΔoxyHb (negative functional response). Following 4.8 s stimulations, more lambs receiving sildenafil or iNO (83% and 100% respectively) showed positive functional response compared to the controls (*p* < 0.05). No significant difference between the three groups was observed at 7.8 s stimulations.

**Conclusion:** In the preterm brain, prolonged somatosensory stimulations increased the incidence of negative functional responses with decreased cerebral oxygenation, suggesting that cerebral oxygen delivery may not match the oxygen demand. Sildenafil and iNO increased the incidence of positive functional responses, potentially enhancing NVC, and cerebral oxygenation.

## 1 Introduction

Neurovascular coupling (NVC) describes the relationship between an increase in cerebral oxygen consumption (primarily driven by increased local neural activity) and a corresponding increase in cerebral oxygen delivery (primarily determined by localised cerebral blood flow (CBF)) (Fox and Raichle, 1986). This response is well characterised in the mature, adult brain and termed ‘functional hyperaemia’ as the increase in CBF always exceeds the increase in cerebral oxygen consumption, thereby ensuring that oxygen supply is always greater than the demand and protecting the brain from hypoxia (Iadecola, 2004). This “positive” cerebral haemodynamic response can be detected by near-infrared spectroscopy (NIRS) as an increase in oxy-haemoglobin (oxyHb) and decrease in deoxy-haemoglobin (deoxyHb), or by functional magnetic resonance imaging (fMRI) as a positive blood oxygen level-dependent (BOLD) signal. Impairment in the cerebral functional haemodynamic response has been identified in several neurological disorders, highlighting the importance of NVC in normal brain function (Girouard and Iadecola, 2006).

In contrast, the cerebral functional response in the preterm or term neonatal brain is variable and can present as positive (similar to the adult response) (Taga et al., 2003; Bartocci et al., 2006), dampened (Colonnese et al., 2008; Arichi et al., 2012) or even “negative” (Kusaka et al., 2004; Nakamura et al., 2017; Inocencio et al., 2021) responses. A “negative” cerebral haemodynamic response is characterised by a decrease, rather than an increase, in cerebral oxyHb. In regards to CBF, a negative functional response can be the result of either an increase in cerebral metabolic rate of oxygen exceeding the increase in CBF or, a decrease in CBF. Regardless, shifts from a positive to a negative functional response can indicate cerebral oxygen supply is insufficient to meet the metabolic demand.

The negative functional response becomes positive with age, and is likely due to the development of the neurovascular unit with growth of its cellular components such as perivascular astrocytes and pericytes, as well as the different time-course of postnatal maturation in the endothelial NO synthase, prostaglandin and adenosine pathways (Harris et al., 2011; Kozberg et al., 2013; Zehendner et al., 2013; Hendrikx et al., 2019; Stackhouse and Mishra, 2021). Immature NVC leading to negative cerebral haemodynamic responses potentially exposes the immature preterm brain to localized cerebral hypoxia repeatedly whenever neuronal activity is increased, thereby contributing to neurological injury often observed in the preterm neonate (Volpe, 2009).

Notably, preterm infants often require extensive treatment in the neonatal intensive care unit (NICU), including vasoactive therapies adopted from adult and paediatric practices with their potential effects on the neonatal brain unknown. For example, persistent pulmonary hypertension (PPHN) is a neonatal condition characterised by high pulmonary vascular resistance restricting pulmonary perfusion and resulting in systemic hypoxaemia. PPHN is commonly treated with inhaled nitric oxide (iNO) or sildenafil administration; both are nitric oxide (NO) modulators to vasodilate the pulmonary circulation and increase blood flow (Martinho et al., 2020). Endogenous NO is a potent vasodilator that induces the conversion of guanosine triphosphate into cyclic guanosine monophosphate, causing arterial smooth muscle relaxation and vasodilation (Ghofrani et al., 2006). In the brain, NO is involved in mediating pressure-flow autoregulation (Bauser-Heaton and Bohlen, 2007) and also carbon dioxide vasoreactivity (Brew et al., 2014). iNO directly increases the level of NO in the lung (Roberts et al., 1997) while sildenafil inhibits the phosphodiesterase-5 enzyme responsible for degradation of cyclic guanosine monophosphate, leading to prolonged vasodilation (Ghofrani et al., 2006).

NO is also a key mediator of NVC (Dormanns et al., 2016). Inhibition of endogenous NO synthase impairs the cerebral haemodynamic response to sensory input (Stefanovic et al., 2007; Hoiland et al., 2020). Both iNO and sildenafil are able to enter the brain (Terpolilli et al., 2012; Gomez-Vallejo et al., 2016). In adults, sildenafil treatment has been shown to improve the cerebral haemodynamic response (Pauls et al., 2018). The effect of NO supplementation and modulation on the cerebral haemodynamic response in the preterm brain is unknown.

Animal models offer the ability to investigate effects of clinical treatments in the developing brain under controlled conditions, without being influenced by the clinical heterogeneity of preterm infants. Using a well-established preterm lamb model (Inocencio et al., 2021) with ratio of white-to-grey matter and pattern of cerebrovascular reactivity similar to the preterm human infant, we aimed to investigate the effect of iNO and sildenafil on the preterm cortical haemodynamic response to somatosensory stimulations using NIRS. We hypothesized that treatment with iNO and sildenafil in clinical dosages used in neonates would increase the baseline CBF and cerebral oxygenation, as well as increase the cerebral haemodynamic response to neural activation.

## 2 Methods and materials

### 2.1 Animal ethics

The Monash Animal Research Platform supplied the pregnant ewes (Merino-Border Leicester cross) used in this study. The study was approved by the Monash Medical Centre Animal Ethics Committee A (2021/02) under guidelines established by the National Health and Medical Research Council of Australia Code of practice for the care and use of animals for scientific purposes.

### 2.2 Ewe preparation and surgery

Preterm lambs were delivered from 13 time-mated twin-bearing pregnant ewes *via* caesarean section at 126-7 days of gestational age. The pregnant ewes were administered betamethasone *via* an intramuscular injection (12 mg; Celestone, Merck Sharp & Dohme, Australia) 48 and 24 h before delivery to accelerate the maturation of the fetal lungs, as used in clinical practice for expected preterm birth (Roberts and Dalziel, 2006). Ewes were fasted for 16 h before surgery with *ad-libitum* access to water. General anaesthesia of the ewe was induced *via* injection of 5% sodium thiopentone (20 mg/kg; Pentothal; Boehringer Ingelheim Australia) into the jugular vein. The ewe was then intubated to allow for positive pressure ventilation (EV500 Anaesthesia Ventilator, Campbell, UCLO Engineering, NSW, Australia). Inhaled isoflurane (1.5%–2.5%) in O_2_ was used to maintain general anaesthesia. Continuous monitoring of the ewe’s end-tidal carbon dioxide (CO_2_) level, heart rate (HR) and oxygen saturation (SpO_2_) was performed during the entire experimental preparation (Surgivet Advisor Vital Signs Monitor, Smiths Medical). Ventilatory settings and fraction of inspired oxygen were adjusted to maintain the ewe’s oxygen saturation (SpO_2_) at 95%–100% and end-tidal CO_2_ between 35–45 mmHg.

### 2.3 Preterm lamb delivery and instrumentation

Surgery was performed under clean but not aseptic conditions as described previously (Inocencio et al., 2021). A maternal midline abdominal incision and hysterotomy were performed, *via* which the fetal head, neck and forelimbs were exteriorized for fetal instrumentation, whilst maintaining the placental-umbilical circulation. An incision was made in the left cubital fossa of the fetal forelimb to expose the median nerve. A silicon cuff with two multi-stranded copper wire electrodes 1 cm apart was placed around the nerve (Inocencio et al., 2021). The fetus was then intubated, lung liquid passively drained and exteriorised onto the maternal abdomen. Ventilation of the preterm lamb was commenced using warm humidified air and volume-guaranteed mode at ∼5 mL/kg (Babylog8000Plus; Draeger; Germany), whilst still maintaining the placental-umbilical circulation. After 1 min of ventilation, the preterm lamb received surfactant *via* the endotracheal tube (100 mg/kg Curosurf; ChiesiPharma; Italy). After 3 min of ventilation, the umbilical cord was clamped and the lamb was transferred to an infant resuscitaire (Fisher and Paykel Healthcare, East Tamaki, New Zealand). The lamb’s body temperature was monitored with a rectal probe and maintained at 39°C–40°C throughout the study. Inhaled isoflurane (1%–1.5%) was used to maintain anaesthesia of the lamb. A pulse oximeter probe (Radical 4, Masimo Frenchs Forest NSW, Australia) was placed on the lamb’s right forelimb to measure SpO_2_. After delivery of both twin lambs, ewes were euthanised using intravenous pentobarbitone (100 mg/kg; pentobarbitone sodium 325 mg/mL, Virbac, Milperra, NSW, Australia).

Following delivery and transition to the infant resuscitaire, a single-lumen catheter (5Fr, 1270.05 polyurethane catheter, Vygon, Paris; France) was inserted retrogradely into one umbilical artery to allow continuous measurement of mean arterial blood pressure (MABP) and heart rate (HR) (DTX Plus Transducer; Becton Dickinson). Umbilical arterial blood samples were collected regularly for measurements of arterial pH, lactate, partial pressures of oxygen (PaO_2_) and carbon dioxide (PaCO_2_) (ABL30; Radiometer, Copenhagen, Denmark). A double-lumen catheter was inserted into the umbilical vein to administer maintenance fluid of 10% glucose (at 3 mL/kg/h) and to administer sildenafil infusion (in lambs allocated to the sildenafil group). Ventilatory settings and fractional inspired oxygen were adjusted to maintain SpO_2_ at >90% and PaCO_2_ at 45–55 mmHg.

### 2.4 Electroencephalography (EEG)

Silver cup electrodes were placed on the shaved scalp of the preterm lamb to record the somatosensory evoked potential (SEP) in bilateral somatosensory cortices (C3-5 and C4-6), according to published coordinates for key landmarks on the sheep’s head (Cook et al., 1987). Two-channel EEG using differential AC amplifiers with bandpass filters set at 1–35 Hz (Grass Instrument Model 79D, Quincy; United States) was used. Depth of anaesthesia was monitored using continuous EEG recording, to avoid the burst-suppression EEG pattern which indicates deep anaesthesia.

### 2.5 Near-infrared spectroscopy (NIRS)

The light emitters and detectors (optodes) of a two-channel Near infrared spectroscopy (NIRO-200, Hamamatsu Photonics, Japan) were placed on the scalp over the bilateral somatosensory cortices, adjacent to the EEG electrodes. For each channel, the emitter and detector were placed 4 cm apart. A light-proof cloth was placed over the optodes to prevent ambient light from interfering with the NIRS recording. The changes in oxygenated haemoglobin (∆oxyHb), deoxygenated haemoglobin (∆deoxyHb) and total haemoglobin (∆total Hb = ∆oxyHb + ∆deoxyHb) in both hemispheres were continuously recorded.

### 2.6 Somatosensory evoked potentials (SEP)

The electrodes implanted around the left median nerve were connected to an isolated constant-current electrical pulse generator (ISOLATOR-11 stimulator isolation unit; Axon Instruments Inc., Foster City, CA, United States) to deliver pre-programmed trains of 2 msec pulses (LabChart 7, ADInstruments Pty Ltd., Bella Vista, NSW, Australia) (Nakamura et al., 2017). The amplitude of the stimulus for each lamb was set to a minimum required to evoke a visible twitch of the left forelimb (∼2–5 mA). Registration of median nerve stimulation as somatosensory stimulation was confirmed by recording the SEP. An electrical pulse was delivered to the median nerve at 1 Hz every 250 ms for over ∼40–80 repetitions and EEG signal from the contralateral cortex was averaged across the 250 ms to produce a SEP response; an example is shown in Supplementary Figure S1. The mean ± SEM latency and amplitude of the first negative or positive component (i.e., N1, P1) in the SEP for all lambs was 62.76 ± 3.55 msec and 15.25 ± 1.57 µV respectively. This is consistent with the first component of the SEP in preterm infants of ∼32 weeks of post-menstrual age occurring approximately 60 msec after transcutaneous stimulation of the median nerve (Tombini et al., 2009).

### 2.7 Experimental protocol and data recording

All physiological signals (MABP, HR, EEG, ∆total Hb, ∆oxyHb, ∆deoxyHb) were recorded digitally at a sampling rate of 1000 Hz using a data acquisition system (Powerlab; ADInstruments, Castle Hill, NSW, Australia) and associated software (LabChart 7, ADInstruments). To elicit and record the cerebral haemodynamic response in each animal, the median nerve was stimulated with stimulus trains of short (1.8 s), medium (4.8 s), or long (7.8 s) durations, using 2 ms pulses at a repetition rate of 3.3 Hz (Nakamura et al., 2017). The beginning of each stimulus train triggered the recording of all physiological signals over a 60 s window, preceded by a 5 s period of pre-stimulus recording baseline to determine the relative magnitude of change of the NIRS signals, MABP and HR following median nerve stimulation. Fifteen to 20 repeats of each stimulus train were delivered to produce a signal-averaged output of all physiological signals (EEG, MABP, HR, and NIRS signals) using Scope software (ADInstruments, Australia). The three stimulus train durations (1.8, 4.8, and 7.8 s) were delivered in random order.

### 2.8 Inhaled nitric oxide/sildenafil treatment

One twin from each pregnancy was randomly allocated to the control group and the other twin allocated to the intervention group. Lambs within the intervention group were randomly allocated to receive either intravenous infusion of sildenafil (Revatio, 10 mg/12.5 mL) or iNO administered *via* the endotracheal tube (INOMAX, Mallinckrodt). The twin lambs were delivered and studied consecutively (Supplementary Figure S2). To account for the potential effect of prolonged anaesthesia, lambs were randomly allocated to either experimental protocol A where the first twin would be allocated as a control, or protocol B where the first twin would be allocated to receiving the intervention (sildenafil or iNO) (Supplementary Figure S2).

Based on neonatal studies on the time required to reach therapeutic plasma levels (Cochius-den Otter et al., 2020), sildenafil infusion at 133 mcg/kg/h (the recommended clinical loading dose) occurred for 1 h before the median nerve stimulations. iNO administration was started at 10 ppm for 10 min, then increased to 20 ppm for 20 min before nerve stimulations, which corresponds to iNO dosage used clinically for the treatment of PPHN (Peliowski et al., 2012). Median nerve stimulation with stimulus train durations of 4.8 and 7.8 s were performed in random order during the sildenafil infusion/iNO inhalation. The 1.8 s stimulation was not conducted in the sildenafil and iNO groups as the mild nature of the short stimulation might not properly reflect the haemodynamic changes induced by the treatments. At the end of the experiment, lambs were euthanised by intravenous injection of pentobarbital at 100 mg/kg.

### 2.9 Data and statistical analysis

The MABP and NIRS data across the 60 s post-stimulus window of recording were averaged every 1 s and expressed as changes (∆) from the averaged 5 s pre-stimulus baseline. Analysis and classification of the NIRS response patterns in the contralateral cortex (right hemisphere) were based on ΔoxyHb, as it presented a more robust signal-to-noise ratio than ΔdeoxyHb as reported previously (Bortfeld et al., 2007; Nakamura et al., 2017). A ‘positive’ or ‘negative’ response was defined as an increase or decrease of oxyHb respectively in the contralateral hemisphere, of more than 2 standard deviations from the pre-stimulus baseline data values.

To assess the duration of the positive cerebral haemodynamic response, we calculated the difference between the times at which the ΔoxyHb reaches its peak amplitude and descends to 40% of the peak amplitude, denoted as T_p40_ (sec) (Supplementary Figure S3).

Statistical analyses were performed using Graphpad Prism 8 (Graphpad Software, La Jolla; United States). Normality was assessed by the Shapiro-Wilk test. The lambs’ weight and, latency and amplitude of SEP were compared between the control, sildenafil and iNO groups using one way ANOVA. Physiological parameters and arterial blood gas measurements were compared between those at the start and the end of the experiment, and between those recorded before and during sildenafil/iNO treatment, using the Student´s paired *t*-test for parametric data or Wilcoxon signed-rank test for non-parametric data. Using the Chi-square test for trend or the Fisher’s Exact test, the proportions of different patterns of haemodynamic response measured by NIRS were compared within the control group between the three stimulation durations, and also compared between the control group and intervention groups (sildenafil or iNO), for 4.8 and 7.8 s stimulations respectively. Effects of the three durations of stimulation on the ΔMABP (peak amplitude and time to peak) and ΔoxyHb responses (peak/nadir amplitude, time to peak/nadir and area under the curve (AUC) in the control group were compared using one-way repeated measures ANOVA, with Tukey’s *post hoc* analysis for parametric data and Friedman test for non-parametric data; for the positive and negative cerebral hemodynamic responses respectively. Pearson correlation analysis was performed to assess relationship between the peak/nadir ΔoxyHb and ΔMABP responses during stimulations. The ΔMABP and ΔoxyHb responses were compared between the control, sildenafil and iNO groups using one way ANOVA, for 4.8 and 7.8 s stimulations respectively. All results are expressed as mean ± SEM, and *p* < 0.05 was considered significant.

## 3 Results

### 3.1 Preterm lamb characteristics

There was no difference between the average weights of preterm lambs allocated to the control (*n* = 11), sildenafil (*n* = 6), and iNO (*n* = 8) groups (Table 1). The total duration for which lambs were subjected to mechanical ventilation post-delivery was ∼2 h. All physiological parameters remained stable for the duration of the experiment and remained within the normal range for preterm lambs under these experimental conditions (Polglase et al., 2005) (Table 1). The latency of SEP was significantly lower in both sildenafil and iNO groups compared to the controls (Table 1; *p* = 0.05 and 0.008 respectively)

**TABLE 1:** Preterm lambs’ (*n* = 11) arterial blood gases, mean arterial blood pressure (MABP), heart rate (HR) and SEP measurements at the beginning and end of the experiment (values are mean ± SEM).

	Contralateral ΔoxyHb response pattern					
	Control		Sildenafil		Inhaled Nitric Oxide	
Parameter	Beginning of Experiment	End of Experiment	Beginning of Experiment	End of Experiment	Beginning of Experiment	End of Experiment
Gender (male/total)	7/11		4/6		6/8	
Weight (kg)	3.3 ± 0.2		3.1 ± 0.3		3.5 ± 0.2	
pH	7.29 ± 0.03	7.33 ± 0.02	7.34 ± 0.04	7.34 ± 0.03	7.29 ± 0.03	7.3 ± 0.05
PaCO_2_ (mmHg)	61.1 ± 4.4	55.8 ± 2.4	55.3 ± 5.2	53.5 ± 3.6	59.0 ± 3.2	53.4 ± 6.6
PaO_2_ (mmHg)	97.1 ± 8.8	99.0 ± 11.5	179.7 ± 53.0	139.7 ± 18.5	78.7 ± 12.7	98.4 ± 1.7
HCO_3_^−^ (mmol.L^−1^)	25.1 ± 0.7	26.6 ± 0.8	26.5 ± 1.1	26.23 ± 1.0	24.8 ± 1.0	23.7 ± 1.8
Base excess (mmol.L^−1^)	1.7 ± 0.9	3.3 ± 1.0	3.0 ± 1.3	2.7 ± 1.2	1.3 ± 1.0	^−^0.9 ± 2.5
Glucose (mmol.L^−1^)	6.1 ± 1.9	6.1 ± 0.9	9.2 ± 2.5	8.1 ± 1.8	3.4 ± 1.7	7.7 ± 1.3
Lactate (mmol.L^-1^)	2.8 ± 0.3	2.7 ± 0.3	2.7 ± 0.7	2.6 ± 0.5	3.3 ± 0.6	3.7 ± 1.2
MABP (mmHg)	59.0 ± 3.2	53.7 ± 3.5	55.0 ± 2.45	53.34± 1.7	68.2 ± 2.5	74.2 ± 15.7
HR (bpm)	165.5 ± 7.6	155.5 ± 10.5	154 ± 7.1	187.1 ± 26.0	192.2 ± 22.8	179.1 ± 15.4
Amplitude of SEP (P/N1) (μV)	13.6 ± 1.4		20.6 ± 5.4		13.5 ± 1.7	
Latency of SEP (P/N1) (ms)	76.4 ± 5.4		51.0 ± 3.4*		52.9 ± 2.5*	

* indicates significant difference to control. *p* < 0.05.

### 3.2 Patterns of cerebral haemodynamic response to somatosensory stimulation in the control group

Based on changes in the oxyHb signal in the contralateral cortex, Table 2 summarises the incidence of positive, negative or no responses, and Table 3 summarises the amplitude and timing of the ΔMABP and ΔoxyHb responses following the three different durations of stimulation.

**TABLE 2:** Contralateral ΔoxyHb response pattern in preterm lambs with and without sildenafil and iNO. Incidence of positive or negative response pattern, based on changes in oxyhaemoglobin (ΔoxyHb) recorded by NIRS from the contralateral hemisphere, in the control, sildenafil and iNO preterm lambs following median nerve stimulation at 3.3 Hz for 1.8, 4.8, or 7.8 s.

Experimental Groups	Duration of Stimulation	No. of Lambs Studied (n)	Positive (n (%)) (males)	Negative (n (%)) (males)	No Response (n (%)) (males)
Control	1.8 sec	11	7 (64) (4)	1 (9)* (1)	3 (27) (2)
	4.8 sec	11	7 (64)# (6)	4 (36) (1)	0
	7.8 sec	11	4 (36) (3)	7 (64) (4)	0
With Sildenafil	4.8 sec	6	5 (83) (3)	1 (17) (1)	0
	7.8 sec	6	5 (83) (3)	1 (17) (1)	0
With iNO	4.8 sec	8	8 (100) (6)	0	0
	7.8 sec	8	5 (63) (4)	3 (37) (2)	0

*p < 0.05 for 1.8 vs. 4.8 vs. 7.8 s in controls,

^#^ *p* < 0.05 for control vs. sildenafil vs. iNO at 4.8 s (chi-square test for trend).

**TABLE 3:** Changes in physiological parameters in preterm lambs with and without sildenafil and iNO. Changes in physiological parameters in preterm lambs with positive or negative contralateral ΔoxyHb response, following median nerve stimulations for 1.8, 4.8, and 7.8 s in the control, sildenafil and iNO preterm lambs (values are mean ± SEM; individual values are shown where n < 3 available).

Experimental Groups	Physiological Parameters		1.8 sec		4.8 sec		7.8 sec	
Control			Negative (n = 1)	Positive (n = 7)	Negative (n= 4)	Positive (n = 7)	Negative (n = 7)	Positive (n = 4)
	ΔMABP	Peak amplitude (mmHg)	0.5	1.1 ± 0.34*	1.2 ± 0.2	2.6 ± 0.5	1.2 ± 0.5	2.0 ± 0.5
		Time to peak (sec)	5	10.3 ± 3.2	10.3 ± 2.6	8.4 ± 0.7	19.3 ± 6.8	10.3 ± 0.6
	Contralateral ΔoxyHb	Peak/ nadir amplitude (μM.cm)	−1.14	2.5 ± 0.4	−6.1 ± 2.3	5.7 ± 1.3	−3.6 ± 0.9	6.3 ± 2.0
		Time to peak/nadir (sec)	2	7.4 ± 2.1	11 ± 2.5	10.9 ± 1.5	14.9 ± 2.1*	13.8 ± 2.5
		AUC (μM.cm.sec)	2.3	19.6 ± 6.5	105.9 ± 25.4	54.4 ± 15.7	57.9 ± 24.2	66.4 ± 21.8
		T_p40_	—	—	6.43 ± 1.62		8.0 ± 1.76	
			1.8 sec		4.8 sec		7.8 sec	
With Sildenafil			—	—	Negative (n = 1)	Positive (n = 5)	Negative (n = 1)	Positive (n = 5)
	ΔMABP	Peak amplitude (mmHg)	—	—	0.5	2.6 ± 0.2	−0.5	2.8 ± 0.8
		Time to peak (sec)	—	—	34	9 ± 0.6	9	11.0 ± 0.3
	Contralateral ΔoxyHb	Peak/ nadir amplitude (μM.cm)	—	—	−5	5.2 ± 0.7	−4.5	5.1 ± 1.5
		Time to peak/nadir (sec)	—	—	24	13 ± 2.3	22	11.6 ± 1.0
		AUC (μM.cm.sec)	—	—	132.7	120.8 ± 25.4	105.2	123.1 ± 57.1
		T_p40_	—	—	22.4 ± 4.5^#^		11.4 ± 5.1	
			1.8 sec		4.8 sec		11.4 ± 5.1	
With iNO			—	—	Negative (n = 0)	Positive (n = 8)	Negative (n = 3)	Positive (n = 5)
	ΔMABP	Peak amplitude (mmHg)	—	—	—	1.8 ± 0.5	0.6 ± 1.0	1.1 ± 0.8
		Time to peak (sec)	—	—	—	7.9 ± 1.0	9.0 ± 0.6	16.0 ± 3.6
	Contralateral ΔoxyHb	Peak/ nadir amplitude (μM.cm)	—	—	—	-	−3.7 ± 0.7	7.5 ± 2.7
		Time to peak/nadir (sec)	—	—	—	10.8 ± 2.6	18 ± 5.1	15.8 ± 3.8
		AUC (μM.cm.sec)	—	—	—	44.3 ± 14.0	75.7± 39.9	75.2 ± 16.5
		T_p40_	—	—	6.5 ± 1.8		5.8 ± 1.5	

Values are shown as mean ± SEM.

* indicates significant difference to 4.8 s,

^#^ indicates significant difference vs. both control and iNO groups (*p* < 0.05).

#### 3.2.1 ΔoxyHb

**FIGURE 1:**
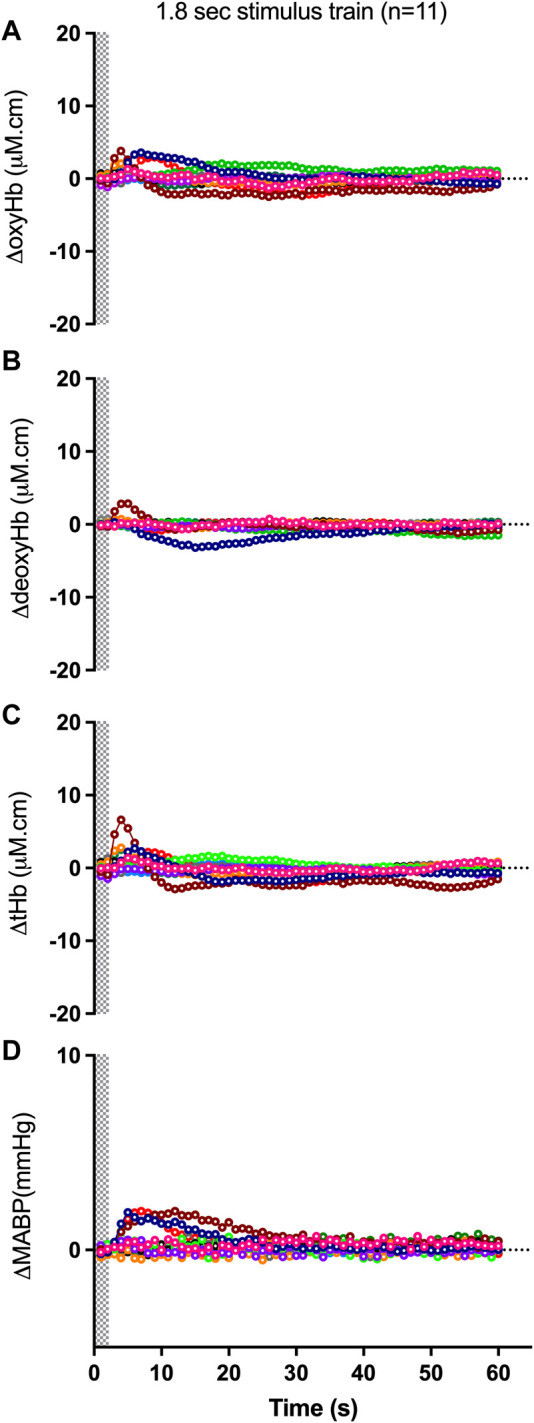
Changes in oxy-, deoxy- and total haemoglobin [ΔoxyHb; **(A)**, ΔdeoxyHb; **(B)** and ∆total Hb; **(C)**], recorded from the contralateral hemispheres, and mean arterial blood pressure [∆MABP; **(D)**], following a 3.3 Hz stimulus train of 1.8 s duration in control preterm lambs. Individual lambs are shown by different colours. Shaded area in each graph indicates the period of stimulation.

**FIGURE 2:**
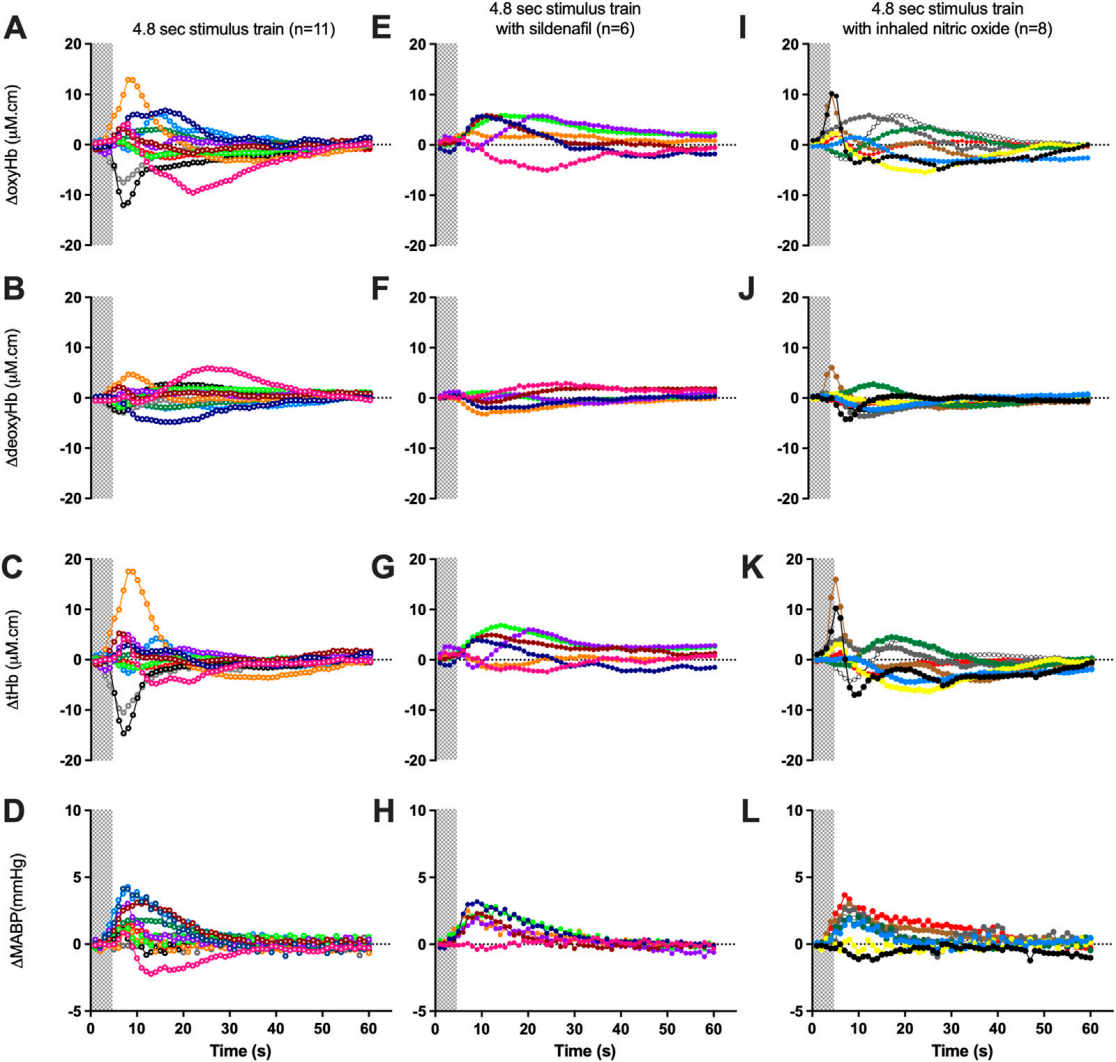
Changes in oxy-, deoxy- and total haemoglobin [ΔoxyHb; **(A, E, I)**, ΔdeoxyHb; **(B, F, J)**, and ∆total Hb; **(C, G, K)**], recorded from the contralateral hemispheres, and mean arterial blood pressure [∆MABP; **(D, H, L)**], following a 3.3 Hz stimulus train of 4.8 s in control lambs (open circles; A**–**D), lambs with sildenafil infusion [solid circles; **(E–H)**] or with inhaled nitric oxide [solid circles; **(I–L)**]. Individual lambs are indicated by different colours. Shaded area in each graph indicates the period of stimulation.

**FIGURE 3:**
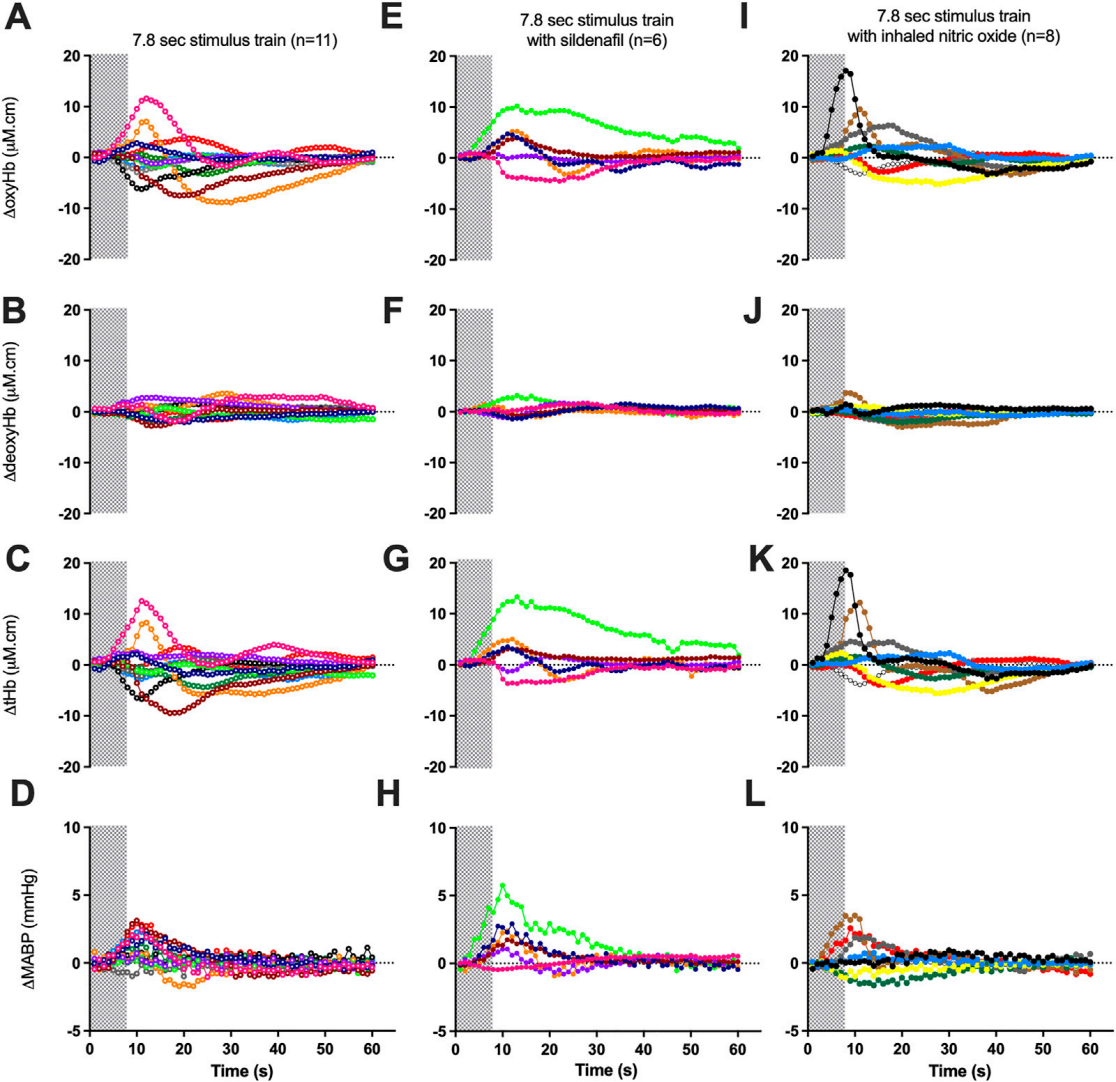
Changes in oxy-, deoxy- and total haemoglobin [ΔoxyHb; **(A, E, I)**, ΔdeoxyHb; **(B, F, J)**, and ∆total Hb; **(C, G, K)**], recorded from the contralateral hemispheres, and mean arterial blood pressure [∆MABP; **(D, H, L)**], following a 3.3 Hz stimulus train of 7.8 s in control lambs [open circles; **(A–D)**], lambs with sildenafil infusion solid circles; **(E−H)**] or with inhaled nitric oxide [solid circles; **(I-L)**]. Individual lambs are indicated by different colours. Shaded area in each graph indicates the period of stimulation.

The proportion of negative responses increased with longer durations of stimulation (*p* = 0.02, Table 2). Following the short (1.8 s) somatosensory stimulation, only one of the 11 control lambs (9%) demonstrated a negative response evident by a decrease in contralateral oxyHb (Table 2; Figure 1A; purple open circles). Seven control lambs (64%) demonstrated a positive cerebral hemodynamic response with an increase in contralateral oxyHb (Table 2; Figure 1A; pink, light green, orange, dark blue, brown, dark green and red open circles), and 3/11 lambs had no change in oxyHb (no response) despite showing similar SEP to all other lambs (Table 2; Figure 1A; black, grey and light blue open circles). Following 4.8 s stimulation, 4/11 (36%) lambs demonstrated negative responses (Table 2; Figure 2A; light green, black, grey and red open circles), and the remaining seven lambs (64%) demonstrated positive responses (Table 2; Figure 2A). Following 7.8 s stimulations, 7/11 (64%) lambs demonstrated negative responses (Table 2; Figure 3A; light green, purple, brown, black, grey, dark green, and light blue open circles), and only four lambs (36%) showed positive changes in the contralateral ΔoxyHb. Only three of the 11 control lambs showed a consistent positive ΔoxyHb response (pink, orange, and dark blue open circles) following all (1.8, 4.8, and 7.8 s) durations of stimulations.

#### 3.2.2 ΔdeoxyHb and Δtotal Hb

Relative to changes in contralateral oxyHb, changes in contralateral deoxyHb were either of very low amplitudes or in the opposite direction to that of the corresponding ΔoxyHb in a majority of lambs (Figure 1B; Figures 2B, 3B). The only exceptions were one lamb at 1.8 and 7.8 s stimulations (Figures 1A, B, 3A, B; brown open circles) and four lambs at 4.8 s stimulations (Figures 2A, B; orange, brown, black, and grey open circles) which displayed concordant response patterns in their ΔoxyHb and ΔdeoxyHb (i.e., both ΔoxyHb and ΔdeoxyHb increased or both decreased). In all lambs, the contralateral ΔtotalHb displayed concordant response patterns to those of their ΔoxyHb across all durations of stimulation.

### 3.3 Changes in blood pressure and cerebral oxyHb after somatosensory stimulation in the control lambs

The peak changes in MABP and peak/nadir changes in ∆oxyHb in the contralateral cortex following median nerve stimulations are shown in Table 3.

The peak amplitude of the ∆MABP was significantly greater following 4.8 s stimulation compared to 1.8 s (*p* < 0.05), in the lambs which showed positive changes in ΔoxyHb. The time taken to reach peak MABP did not differ between the stimulation durations (Table 3).

The amplitude of peak/nadir change in the ∆oxyHb was not different between different durations of stimulation for the positive or negative responses respectively. The time taken to reach the nadir ∆oxyHb was significantly greater following 7.8 s stimulations compared to 4.8 s (*p* < 0.05) for the negative responses. The time taken to reach the peak ∆oxyHb was not different (Table 3). The contralateral ΔoxyHb responses showed no correlation with the peak change in MABP following all durations of stimulation, suggesting that the blood pressure change is not the determinant of the cerebral haemodynamic response (Supplementary Figures S4A–S4C).

### 3.4 The effects of sildenafil and inhaled nitric oxide treatment

Arterial blood gases, MABP, HR, SEP, and NIRS data obtained in preterm lambs at the start and after 60 min of sildenafil treatment or after 30 min of iNO inhalation are summarised in Table 4. Following administration of sildenafil or iNO, there were no significant changes in the baseline physiological or cerebral haemodynamic parameters.

**TABLE 4:** Arterial blood gases, mean arterial blood pressure (MABP), heart rate (HR) together with EEG and NIRS data of preterm lambs at the start and after 60 min of sildenafil infusion (*n* = 6) or 30 min of iNO inhalation (*n* = 8) (values are mean ± SEM).

	Sildenafil		iNO	
	Start of infusion	After 60 min of infusion	Start of inhalation	After 30 min of inhalation
pH	7.34 ± 0.02	7.34 ± 0.03	7.30 ± 0.03	7.29 ± 0.05
PaCO_2_ (mmHg)	63.8 ± 2.8	53.23 ± 4.0	56.0 ± 3.5	55.7 ± 6.9
PaO_2_ (mmHg)	166.5 ± 46.0	147.72 ± 19.2	77.2 ± 13.6	105.2 ± 16.7
HCO_3_^−^ (mmolL^−1^)	25.3 ± 0.9	26.32 ± 1.0	24.5 ± 1.2	23.4 ± 2.0
Base excess (mmolL^−1^)	2.1 ± 1.2	2.65 ± 1.2	0.7 ± 1.4	−0.4 ± 2.6
Glucose (mmolL^−1^)	4.0 ± 1.1	9.5 ± 2.4	5.2 ± 2.2	7.6 ± 1.9
Lactate (mmolL^−1^)	2.7 ± 0.6	2.6 ± 0.5	3.4 ± 0.7	3.7 ± 1.2
MABP (mmHg)	61.3 ± 4.8	54.4 ± 2.0	65.0 ± 3.2	59.7 ± 2.3
HR (bpm)	156.5 ± 9.5	167.8 ± 13.7	189.2 ± 26.2	176.1 ± 9.5
∆oxyHb (μM.cm)	−13.9 ± 7.9	−41.5 ± 82.3	−7.2 ± 6.8	−22.2 ± 31.0
∆deoxyHb (μM.cm)	−11.8 ± 10.0	−25.2 ± 72.8	−3.5 ± 5.6	12.0 ± 33.6
∆Total Hb (μM.cm)	−2.1 ± 5.2	−16.3 ± 24.0	−3.7 ± 8.3	−34.2 ± 15.9

### 3.5 Responses to nerve stimulation with sildenafil and inhaled nitric oxide treatment

Overall, the proportions of positive responses were higher in both intervention groups compared to the control groups, and reached significance with the 4.8 s stimulation (*p* < 0.05, Table 2).

During sildenafil infusion, five of the six lambs (83%) demonstrated a consistently positive ∆oxyHb response after both 4.8 and 7.8 s stimulations (Table 2; Figure 2E). Only one lamb (Figure 2E; pink circles) demonstrated a consistently negative response.

During iNO inhalation, all eight lambs demonstrated a positive ∆oxyHb response after 4.8 s stimulation (Table 2; Figure 2I). Following 7.8 s stimulation, five out of the eight lambs (63%) retained a positive ∆oxyHb response and three had a negative ∆oxyHb response (Table 2; Figure 3I: negative response—red, white and yellow circles).

The T_p40_, which assesses the temporal characteristics of the positive ΔoxyHb response was significantly higher in lambs treated with sildenafil compared to both control and iNO groups (*p* = 0.04 and 0.03 respectively), following 4.8 s stimulations (Table 3). There was no difference in T_p40_ between the three groups in response to 7.8 s stimulations.

There were no differences in the amplitude of or time to reach peak ΔMABP and ΔoxyHb between the 4.8 and 7.8 s stimulations within the sildenafil or iNO group; or between the three groups of lambs (control, sildenafil, and iNO) at 4.8 and 7.8 s stimulations respectively (Table 3).

## 4 Discussion

Endogenous NO is a key mediator in NVC (Dormanns et al., 2016). Our study is the first to address the effects of NO modulating therapies on the cerebral functional haemodynamic response in the preterm brain. The key observations are that preterm lambs without NO treatment showed increasing incidence of negative cerebral haemodynamic responses with longer durations of somatosensory stimulation (from 9% following the 1.8 s stimulation, to 36% with the 4.8 s, and 64% with the 7.8 s stimulations, Table 2). More preterm lambs receiving sildenafil and iNO showed positive responses after prolonged somatosensory stimulations, suggesting that both NO modulators enhanced the cerebral oxygen delivery relative to consumption.

### 4.1 Increased incidence of negative cerebral haemodynamic responses with prolonged somatosensory stimulations

Consistent with the results of this study, we have previously reported a similar increase in incidence of negative functional responses following prolonged somatosensory stimulations in fetal lambs of the same gestational age (Nakamura et al., 2017). Negative cerebral responses have also been observed in neonatal rodents (Kozberg et al., 2013; Zehendner et al., 2013) and human neonates (Bartocci et al., 2001; Kusaka et al., 2004; Zimmermann et al., 2012). The negative response involved reductions in both oxyHb and total Hb, and likely arose from arterial vasoconstriction. In neonatal rats, hindpaw stimulations also produce a negative haemodynamic response with global cerebral vasoconstriction, but conversely produces a positive response with pial artery dilatation in the adults rats (Kozberg et al., 2013), suggesting the development of functional hyperaemia with increasing age. In addition, prolonged somatosensory stimulations drive a decline in EEG activity and reduce CBF in the neonatal mouse (Zehendner et al., 2013), suggesting the inability to sustain neural processing during prolonged stimulations in the immature brain. The decrease in CBF may also be due to redistribution and hence ‘haemodynamic steal’ of blood towards an adjacent cortical region of neuronal activity (Kozberg and Hillman, 2016; Suarez et al., 2021). In contrast, prolonged stimulation in adult mice was associated with stable neural processing and increased regional CBF. Taken together, the resulting negative functional responses suggests a potential for regional cerebral hypoxia to develop in the preterm brain when neuronal activation is prolonged.

### 4.2 Sildenafil/iNO treatment

#### 4.2.1 Resting state

The 60 min of sildenafil infusion or 30 min of iNO did not change the cerebral oxyHb and total Hb, indicating that both sildenafil and iNO does not alter baseline cerebral perfusion. As NO modulators, both sildenafil and iNO can lead to cerebral vasodilation with increased ΔoxyHb and ∆tHb. However, the concurrent pulmonary vasodilation induced by sildenafil and iNO may lead to higher PaO_2_ and can result in cerebral vasoconstriction and reduced CBF (Day, 2001; Polglase et al., 2016), thereby negating any increases in the cerebral oxyHb and total Hb. Nonetheless, the PaO_2_ increase following sildenafil and iNO administration in our lambs were small and insignificant. Our findings align with previous studies in adults which show that sildenafil treatment does not change the overall CBF (Diomedi et al., 2005; Rosengarten et al., 2006; Al-Amran et al., 2012; Jahshan et al., 2017; Lindberg et al., 2017). However, in contrast to studies of rat pups (Charriaut-Marlangue et al., 2012) or piglets (Pastor et al., 2019) with induced brain injury in which iNO treatment leads to cerebral vasodilation and restores CBF, our preterm lambs (without prior brain injury) did not show any significant change of cerebral haemodynamics following iNO. Possibly, iNO may have selective actions depending on the presence of brain injury.

#### 4.2.2 During somatosensory stimulations

During the administration of sildenafil or iNO, more preterm lambs demonstrated a positive functional response with increased ∆oxyHb following nerve stimulations, compared to the control lambs (Table 2). Sildenafil is reported to enhance the cerebral haemodynamic response to auditory and visual stimulations in adult humans (Schultheiss et al., 2001; Lindberg et al., 2017). Studies of other phosphodiesterase-5 inhibitors in adults also report improved responsiveness of the cerebral vasculature, particularly in disease states associated with impaired cerebral vasoreactivity (Pauls et al., 2018). In our preterm lambs, sildenafil reduced the incidence of negative functional response and cerebral vasoconstriction following neuronal stimulation as found in the control lambs. The ∆oxyHb responses were also sustained for longer (higher T_p40_) in the sildenafil group compared to both the control and iNO groups following 4.8 s stimulations, consistent with reduced breakdown of cyclic guanosine monophosphate leading to prolonged vasodilation and cerebral haemodynamic response. There was no group difference in the T_p40_ following 7.8 s stimulations, suggesting that the effect of sildenafil in sustaining the functional hyperaemia may not apply to more prolonged neuronal activities.

To our knowledge, the vital role of NO in augmenting functional hyperaemia have only been demonstrated using inhibition of endogenous NO (Stefanovic et al., 2007; Hoiland et al., 2020). Our study is the first to show that exogenous NO supplementation has no adverse effect on, and may potentially promote the cerebral functional response. The effect of iNO in inducing the positive functional response appeared more marked following moderate (4.8 s) compared to prolonged (7.8 s) stimulations (100% vs. 63% positive response), suggesting that iNO may have limited capacity to ameliorate the negative response following prolonged neuronal stimulations. Notably, the cerebral effect of iNO in preterm infants warrants caution. While several studies have reported the use of iNO in preterm infants decreases the risk of intraventricular haemorrhages (Kinsella et al., 2006), cerebral palsy (Tanaka et al., 2007) and neurodevelopmental disabilities (Mestan et al., 2005), early use of iNO during the first 3 days of life shows a trend towards increased intraventricular haemorrhaging (Barrington et al., 2017).

The SEP in our preterm lambs showed very similar morphology to that recorded using transcutaneous stimulation of the median nerve in term human infants, with a N1/P1 latency similar to that in preterm infants of ∼32 weeks of post-menstrual age (Tombini et al., 2009). Compared to the control lambs, the latency of SEP was lower in lambs with sildenafil or iNO. Previous literature suggests that SEP latency becomes shorter and its amplitude greater with increasing post-menstrual age in preterm infants, correlating with the maturation of myelination, development of thalamo-cortical connections and synapses (Vanhatalo and Lauronen, 2006; Tombini et al., 2009). Interestingly, sildenafil has been reported to enhance event- or task-related EEG potentials in healthy adults (Schultheiss et al., 2001). Moreover, inhibition of nitric oxide synthase attenuates the cortical SEP as well as the cerebrovascular functional response during somatosensory stimulation (Ngai et al., 1995; Ngai et al., 1998). Our finding supports the potential of sildenafil and iNO to enhance neurotransmission in the preterm brain.

#### 4.2.3 Clinical implications

Sildenafil and iNO reduced the incidence of negative responses in our preterm lambs, while sildenafil also sustained the positive oxyHb response for longer durations. It is not clear whether the preterm cerebral functional response affect or correlate with cognitive performance or neuropathy. Notably, in adults, the magnitude of the cerebral functional response correlates positively with cognitive function in normal (Sorond et al., 2013) and disease states (Girouard and Iadecola, 2006). Sildenafil augments the adult cerebral functional response (Pauls et al., 2018), enhances task performance in healthy participants (Grass et al., 2001), and leads to functional benefits in patients with cerebral infarct (Schlindwein et al., 2010) and spastic quadriplegia (Cocchiarella, 2012). In neonatal rodents with brain injury, sildenafil demonstrates anti-apoptotic and anti-inflammatory properties, and promotes angiogenesis and neurogenesis (Zinni et al., 2021), but the mechanisms remain unclear. Our results suggest that improved cerebral functional response is a potential pathway underlying the neuroprotective actions of sildenafil in the immature brain. Clinical trials being conducted by Wintermark and colleagues to evaluate sildenafil in treating term asphyxiated newborns and neonatal encephalopathy are currently ongoing (NCT02812433 and NCT04169191). NIRS measurement of the cerebral functional response may be a useful biomarker for monitoring new interventions in infants. However, enhancement of cerebral haemodynamic response by NO modulators may pose the risk of localized hyperperfusion and hyperoxia, which warrants caution in extremely preterm infants especially during the early postnatal days due to the risk of intraventricular haemorrhages.

## 5 Limitations

The experiments were performed during the first 2h of postnatal life in late preterm lambs. It remains unknown if the enhancement in cerebral functional response by NO modulators will be affected by the postnatal developmental changes, or gestational age at birth. Our pregnant ewes received antenatal betamethasone, as current data shows that more than 90% of preterm infants born before 34 weeks of gestation were exposed to one or more doses of antenatal corticosteroids. Notably, maternal betamethasone has been reported to affect the resistance of fetal cerebral arteries, and hence may affect the cerebrovascular response (Vafaei et al., 2021). Sildenafil and iNO are used to treat PPHN, however our lambs did not undergo echocardiography to evaluate the pulmonary pressure. Further studies are required to investigate if our observations would apply in neonates with PPHN and receiving sildenafil/iNO treatment. CBF is increased by isoflurane at doses above 1.6% (Eger, 1984). Accordingly, we limited the concentration of isoflurane to <1.5% and continuously monitored the EEG to ensure the absence of the burst suppression pattern indicative of deep anaesthesia. To assess components of the neurovascular unit, we have collected the preterm lamb brains for further histopathological and molecular analyses which will be the subject of another manuscript. Further studies are also required to investigate the role of the nitric oxide (NO)-cyclic guanosine monophosphate (cGMP)-protein kinase G (PKG) pathway in the preterm neurovascular coupling.

## 6 Conclusion

The effects of NICU therapies on the preterm cerebral functional haemodynamic response remain largely unexplored. Sildenafil and iNO increased the incidence of positive haemodynamic responses in the preterm brain during prolonged somatosensory stimulations, suggesting the potential of NO modulating therapies to enhance cerebral oxygen delivery relative to consumption. The effects appeared more marked with sildenafil which also sustained the duration of the positive cerebral haemodynamic response. Our work has provided new information on sildenafil-induced augmentation of cerebral functional response in the preterm brain.

## Data Availability

The raw data supporting the conclusion of this article will be made available by the authors, without undue reservation.
