# Supplementary material for: Cerebral haemodynamic response to somatosensory stimulation in preterm lambs is enhanced following sildenafil and inhaled nitric oxide administration
Source: Front Physiol. 2023 Jan 25;14:1101647. doi: 10.3389/fphys.2023.1101647 (PMC9905131; doi:10.3389/fphys.2023.1101647)
Supplement: Supplementary file 1 [file DataSheet1.docx]

**Supplementary Tables and Figures**

**Supplementary Figure 1.** Example of the somatosensory evoked potential (SEP) in a control preterm lambs recorded from the contralateral cortex, in response to median nerve stimulation at 1 Hz for 40 sec.

******

**Supplementary Figure 2.** Each pair of twin preterm lambs were randomly allocated to the experimental protocol of either (A) or (B). One twin was then randomly allocated to receive either sildenafil or iNO.

**Supplemental Figure 3.** Illustrative figure for calculating the time taken for a positive cerebral haemodynamic response to fall, denoted as T_p40_. In this example, the ΔoxyHb reaches its peak amplitude of 5.9 μM.cm at 15 sec following the median nerve stimulation (shaded area), and descends to 40% of the peak amplitude (2.3 μM.cm) at 45 sec. Tp40 indicates the duration between these 2 time points and is 30 sec.

**Supplemental Figure 4.** Correlations of peak changes in oxyhaemoglobin (ΔoxyHb) recorded from the contralateral hemisphere for the positive response pattern, or the nadir ΔoxyHb for the negative response pattern, with the peak changes in mean arterial blood pressure (∆MABP) following 3.3 Hz stimulus trains of 1.8 (A), 4.8 (B) and 7.8 (C) sec duration in the control lambs. Individual lambs (n=11) are shown by different colours. No significant correlation was found between ∆oxyHb and ∆MABP with all 3 durations of stimulation.
